# Pregnant women follow‐up service, Shewa, Ethiopia

**DOI:** 10.1002/hsr2.561

**Published:** 2022-03-22

**Authors:** Dawit Baye Haile, Aragaw Eshetie Aguade, Moges Zerihun Fetene

**Affiliations:** ^1^ Department of Statistics, College of Natural and Computational Science Dilla University Dilla Ethiopia; ^2^ Department of Statistics, College of Natural and Computational Science University of Gondar Gondar Ethiopia

**Keywords:** Cox‐PH model, Kaplan–Meier estimator, pregnant women, time to onset of pre‐eclampsia

## Abstract

**Background and Aims:**

The goal of this study was to demonstrate the effects of factors related with time to developing pre‐eclampsia (PE) among pregnant women follow‐up service at Arerti Primary Hospital.

**Methods:**

A survival analysis was employed on a pregnant women's follow‐up service from September 2018 to June 2019 at the Arerti Primary Hospital. A closed‐form sample size formula for estimating the effect of the time‐to‐event data was used. Both the descriptive method and Cox proportional hazards model were applied to compute the research survival data.

**Results:**

Using the Kaplan–Meier estimation technique, the univariable analysis shows that the survival time median is 7 months and 3 weeks. The graph of Kaplan–Meier estimate of total survival functions indicates a decreasing pattern of survivorship function. We used the Kaplan–Meier estimates to investigate the effects of observed differences among different categories of the factors, we applied the Log‐rank test. The final survival model outcomes weight, marital status, age, history of PE, and multiplicity were related to a substantial hazard of evolving PE.

**Conclusion:**

On the basis of our final survival model results, we recommended that all pregnant women having such risk factors should see a health care professional and control their medical condition before and during pregnancy. Advising women about proper body weight in each follow‐up period is supported. Finally, health experts should advise pregnant women about potential risk factors related to PE.

## 1 INTRODUCTION

Maternal mortality is the death of a woman due to pregnancy and childbirth.^1^ Maternal mortality is the death of a woman while pregnant or within 42 days of the pregnancy's termination. This maternal death is caused by complications either directly or indirectly related to the pregnancy, but not from accidental or incidental causes during pregnancy and childbirth.^2^

Pre‐eclampsia (PE) is a leading cause of maternal mortality and morbidity worldwide.^3^ Although the cause of PE remains largely unclear, evidence suggests that several factors including, extremes of age, weight, family history of PE, history of PE in a previous pregnancy, parity, type of pregnancy (single or multiple), family history of diabetes mellitus and hypertension are determinant risk factor related to the condition.^4^, ^5^, ^6^, ^7^

Worldwide, maternal mortality fell from 342 to 211 maternal deaths per 100,000 live births during the period 2000–2017. In 2017, two‐thirds of all maternal deaths occurred in sub‐Saharan Africa, where the maternal mortality ratio was 542 deaths per 100,000 live births. Although this region has achieved significant progress in lowering maternal mortality since 2000, maternal mortality is still almost 78 times higher than in Australia and New Zealand, which has the lowest ratio of any region. Major efforts are needed to bring maternal mortality under 70 deaths per 100,000 live births by 2030, as prescribed by Sustainable Development Goals 3.1.^8^

PE remains a global problem.^9^ It is common problem in developing countries because of illiteracy, poor antenatal care (ANC) follow‐up, and lack of health awareness.^10^ In Ethiopia access to health care is limited, PE is a leading cause of maternal mortality, with estimates of 16% maternal deaths per year.^11^ Despite preventive measures such as the expansion of health facilities, maternal waiting homes, and training of health professionals are undertaken by the government of Ethiopia to reduce maternal and perinatal mortality, maternal and perinatal mortality related to PE is still on an increase.^12^, ^13^, ^14^ This indicates that there are several risk factors associated with their occurrence and the survival of this disease. Therefore, this study attempted on assessing the risk factors on time to develop PE among pregnant women follow‐up service.

## 2 METHODS

### 2.1 Study area

The research area is located in Minjar Shenkora district in North Shewa, Ethiopia. This town is found 132 km away from the capital city of Ethiopia, Addis Ababa.

### 2.2 Study design

A retroactive follow‐up study of pregnant women was implemented at Arerti Primary Hospital between September 2018 and June 2019. The survival data were taken from the pregnant women's follow‐up charts which contain important factors and covariates.

### 2.3 Study population

The study population covered all the pregnant women who followed up in the given time period at Arerti Primary Hospital. The pilot survey was conducted to determine a suitable hypothesized sample size.

A systematic probability sampling technique was implemented for choosing a desirable sample size listed in the sampling frame of pregnant women cards which contains their coded numbers and names. From a total of pregnant women followed up in the survey time, the random interval (*K*) was computed by allocating the total pregnant women followed up in the ward from September 2018 to June 2019. The value of *K* can be computed by dividing the total number of participants who get service in the hospital during the study time period by the total sample size which equals 5, that is, *K* = 1220/244 = 5. A random number was selected from one to five by using a random table and in each fifth pregnant woman followed up in the ward, we selected the participants.

### 2.4 Inclusion and exclusion criteria

All pregnant women on ANC follow‐up service at the booking visit who have two and more than two visits were extracted and followed up until delivery or development of the outcome included in the study.

### 2.5 The outcome variable

The response variable for this study is time to develop PE during the follow‐up period of pregnant women attending ANC service at Arerti Primary Hospital between September 2018 and June 2019.

### 2.6 Method of data analysis

Survival data analysis was employed to address the aim of this study. The analysis involves; descriptive statistics which include survival distribution and Kaplan–Meier survival functions estimation which is used for the estimation of the time to development of PE from a sample and semiparametric method analysis uses both univariable and multivariable Cox proportional hazards models. The researcher was applied R software version 3.2.3.

### 2.7 Ethics and consent

The University of Gondar committee on the research initially granted permission to conduct the study. Second, it has permission from the medical director's office of Arerti Primary Hospital to study the research. To ensure the participants' anonymity, the data collector has not related their names and identity numbers with the respondents' information. Furthermore, all collected data were treated as confidential and were not used beyond the scope of the study.

## 3 RESULTS

### 3.1 Descriptive analysis

The study included 201 pregnant women who followed in the ANC service at Arerti Primary Hospital on the study period September 2018 to June 2019.

The descriptive results of the study indicate that out of the total pregnant women who were examined, more than 80% of the women had no consecutive previous abortion. The median age and the weight of pregnant women were above 25 years and 50 kg, correspondingly. Concerning the prior information of PE, more than 85% of the pregnant women had no prior history of PE. Majority of the pregnant women, more than 85% of them were married and more than 10% of pregnant women were unmarried; and out of married women, more than 98% of them were censored, the remaining of them were event occurred, and out of unmarried women more than 41% of them were censored and the remaining were event occurred. Regarding the current pregnancy women status, more than 85% of them were singleton pregnancy and the remaining of them were twin; and among singleton pregnant women more than 95% of them were censored, the remaining were event occurred, and from the total of twin pregnant women more than 46% were censored, the remaining were event‐occurred.

### 3.2 Result of survival data analysis

The total follow‐up period of pregnant women was 10 months. The median survival time of pregnant women was 7 months and 3 weeks. At the final stage, more than 19% of pregnant women developed PE. We plotted the Kaplan–Meier survivor functions for the categorical covariates to look at the arrangements of survival practice of the covariates included in the study. The Kaplan–Meier survival plots showed the pattern of one survivorship function lying overhead another, indicating the class explained by the upper curve had a good survival probability than the lower curve.

### 3.3 Results of the log‐rank test

To investigate the significance of the observed difference in the Kaplan–Meier estimates of the survivor functions among different categories of the factors, we applied the log‐rank test. The log‐rank test result indicated that there were substantial differences in survival probability of pregnant women in covariates of marital status, abortion, diabetes, PE, substance, multiplicity, gravidity, and parity at 5% of the significance level.

### 3.4 Cox proportional hazards (PH) model

The classical point of view was used to select the variables to be included in the survival process. That is, to determine the variables to be included in the survival model, an automatic variable selection method was used. Then, the command revealed that the variables age, weight, PE, parity, multiplicity, and marital status were considered as candidate independent variables to be included in event times.

The final fitted model was the Cox‐PH regression model which indicates the influences related to an important hazard of developing PE through the gestational follow‐up time were multiplicity, age, marital status weight, and PE. When we see multiple pregnancies of women, singleton pregnant had less risk of developing PE compared to twin pregnant. Women with no previous history of PE had a 7% less risk of developing PE as compared to women with a previous history of PE. In the case of marital status, the predictable hazards of PE for married pregnant women compared to those unmarried pregnant women were less than two times. This indicates that unmarried pregnant women were highly at risk to develop PE when we compared those married women. Regarding the age of pregnant women, the hazard of developing PE was increased, when the age of pregnant women is increased. This indicates that those older pregnant women were highly at risk to develop PE. For weight, by keeping other variables constant, those pregnant women having less weight have fewer hazards to develop PE, which indicates that those pregnant women who have excess weight are associated with a high risk to develop PE.

### 3.5 Proportional hazard assumption checking

The proportional hazards assumption asserts that the hazard ratios are constant over time and it is important to use a fitted proportional hazards model. The risk of developing PE must be the same no matter how long subjects have been followed. To test this assumption, using the Cox‐PH function in R, scaled Schoenfeld residual tests are used. The hypothesis of no correlation is tested using the chi‐square test statistic. For this case Table 1, all covariates are insignificant with (*p* > 0.05) and the Global test is not significant indicates that the PH assumptions were met.

**Table 1:** Global test for proportionality assumption

Covariates	*χ* ^2^	*p*
Age	0.569	0.451
Weight	1.58	0.209
Pre‐eclampsia	4.27	0.390
Multiplicity	3.20	0.074
Marital Status	2.81	0.094
Parity	3.41	0.890
Global	0.169	0.078

**Figure 1:**
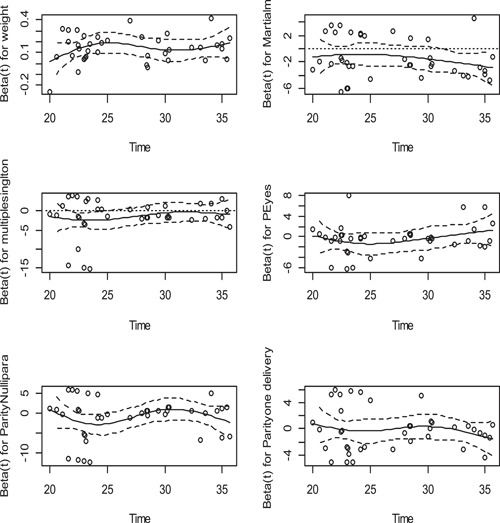
Plot of scaled Schoenfeld residual for test of proportionality assumption

Figure 1 shows that the scaled Schoenfeld residuals are randomly distributed and a smoothened curve does not exhibit much departure from the horizontal line suggesting that the proportional hazards assumption was not violated.

## 4 DISCUSSION

The main aim of this study was to evaluate factors that are related to survival time/time to PE at Arerti Primary Hospital. Thus, to answer the aims of the study we applied the Cox proportional hazards model, for time to PE for both covariates and factors. The survival time was considered by the Cox Proportional model.^15^ From the final multivariable Cox model, we have found that age, weight, PE, the multiplicity of pregnancy, and marital status of pregnant women were significant at 5% level significance coinciding with the study done by Florindo et al.^16^

PE is one of the major causes of maternal mortality worldwide. At the final stage, more than 19% of pregnant women developed PE. All women who had a prior history of PE and weight of gestational period had a meaningfully higher hazard to develop PE. When we see the previous history of PE and baseline weight, pregnant women who have prior history of PE and weight of gestation had a meaningfully higher hazard of developing PE through the gestational follow‐up period. The association of maternal previous history of PE and risk of PE was found to be significant. The association of maternal weight and risk of PE was found to be significant. This is supported by the studies done by Musa et al.^17^ and Hutcheon et al.^18^ Regarding maternal age, in this study, the relationship of risk of PE and maternal age had significant. The results are consistent with the study done by Baley and Wudad.^19^ This study showed that marital status had a statistically significant association with the development of PE. This study results coincide with the study done by Tessema et al.^20^

## 5 CONCLUSION

From the result of this study, we get that there were different influences associated with PE. Using the Cox‐PH model, the covariates of age, weight, previous history of PE, the multiplicity of pregnancy, and marital status were found to be statistically significant effects for time to PE. Therefore, all pregnant women having such risk factors should be seeing a health care professional and control their medical condition before and during pregnancy. Advising women about proper body weight in each follow‐up period is supported. Finally, we recommend that health experts will advise pregnant women about potential risk factors related to PE. Further studies shall be done on PE in a well‐controlled manner and using an advanced methodology.

## CONFLICTS OF INTEREST

The authors declare no conflicts of interest.

## AUTHOR CONTRIBUTIONS

**Dawit Baye Haile**: Conceptualization, Data curation, Formal Analysis, Methodology, and Writing – original draft; **Aragaw Eshetie Aguade**: Methodology, Supervision, and Writing – review and editing; **Moges Zerihun Fetene**: Supervision, Writing – review and editing, and manuscript formulation. All authors of the paper carefully read and approved the final version of the manuscript. Dawit Baye Haile had full access to all of the data in this study and takes complete responsibility for the integrity of the data and the accuracy of the data analysis.

## Data Availability

The authors confirm that the data supporting the findings of this study are available from the first author on reasonable request.
